# Solid Pseudopapillary Neoplasm of the Pancreas in Young Male Patients: Three Case Reports

**DOI:** 10.1155/2017/9071678

**Published:** 2017-01-31

**Authors:** Akira Aso, Eikichi Ihara, Kazuhiko Nakamura, Irina Sudovykh, Tetsuhide Ito, Masafumi Nakamura, Tetsuo Ikeda, Nobuyoshi Takizawa, Yoshinao Oda, Shuji Shimizu

**Affiliations:** ^1^International Medical Department, Kyushu University Hospital, Fukuoka, Japan; ^2^Department of Medicine and Bioregulatory Science, Graduate School of Medical Sciences, Kyushu University, Fukuoka, Japan; ^3^Endoscopic Therapeutics Department, Kyushu University Hospital, Fukuoka, Japan; ^4^Department of Surgery and Oncology, Graduate School of Medical Sciences, Kyushu University, Fukuoka, Japan; ^5^Department of Surgery and Science, Graduate School of Medical Sciences, Kyushu University, Fukuoka, Japan; ^6^Department of Anatomic Pathology, Graduate School of Medical Sciences, Kyushu University, Fukuoka, Japan

## Abstract

A preoperative diagnosis of solid pseudopapillary neoplasms (SPNs) in young male patients is difficult to achieve using radiological images. We herein present three cases of young male patients with relatively small SPNs. Endoscopic ultrasound (EUS) showed well-encapsulated, smooth-surfaced, heterogeneous solid lesions in all patients, and all preoperative diagnoses were achieved by EUS-guided fine needle aspiration (EUS-FNA). The final pathological diagnosis after surgery was an SPN with a Ki-67 labeling index of <2%. SPNs should be considered even in young male patients. EUS with EUS-FNA could be a useful diagnostic modality for SPNs even in young male patients.

## 1. Introduction

Solid pseudopapillary neoplasms (SPNs) are rare, low-grade, epithelial neoplasms that are usually discovered incidentally in young female patients [1, 2]. A preoperative diagnosis is difficult to achieve because the clinical imaging findings of SPNs are vary widely. Some SPNs present as solid tumors, while others are observed as cystic lesions and are therefore usually diagnosed postoperatively. SPNs in young male patients are extremely rare. Because of the limited information for such patients, it is currently difficult to predict the likelihood of SPNs in young males. Additionally, SPNs seem to have higher malignant potential in male than in female patients [3, 4]. Thus, it is important to accumulate clinical information about SPNs in young male patients. We herein present three cases of SPNs in young male patients.

## 2. Patients and Methods

A total of 4867 patients underwent endoscopic ultrasound (EUS) at Kyushu University Hospital from April 2009 to June 2015. We retrospectively reviewed the clinical data of these patients. Among them, only three male patients were diagnosed with SPNs, and we present these three cases in this report. The SPNs were preoperatively diagnosed by EUS-guided fine needle aspiration (EUS-FNA) using a curvilinear echoendoscope (GF-UCT240-AL5; Olympus Medical Systems Co., Tokyo, Japan) through the stomach wall with a 22-gauge EchoTip Ultra needle (Wilson-Cook Medical Inc., Tokyo, Japan). After examination by on-site cytopathologists to evaluate the sample quality, the final diagnosis was determined by an experienced histopathologist at Kyushu University.

## 3. Case Reports

### 3.1. Case  1

**Figure 1:**
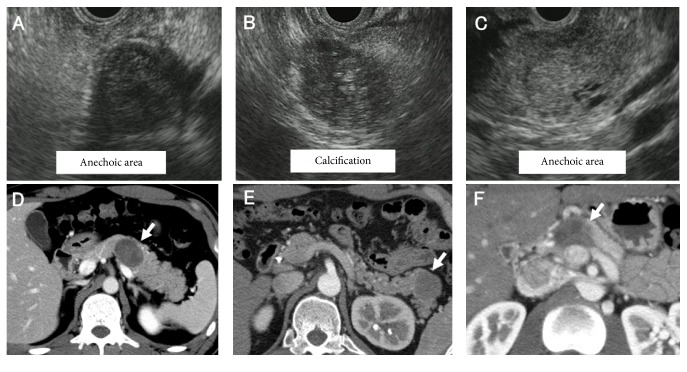
Endoscopic ultrasound and computed tomography findings of three young male patients with solid pseudopapillary neoplasms. The endoscopic ultrasound and computed tomography findings of three young male patients with solid pseudopapillary neoplasms are shown. Solid and hypoechoic masses with anechoic areas were observed in (A) Case  1 and (C) Case  3. Internal calcification was observed in (B) Case  2 by endoscopic ultrasound. Computed tomography showed low-density tumors with slight enhancement at the margin in all three cases.

**Figure 2:**
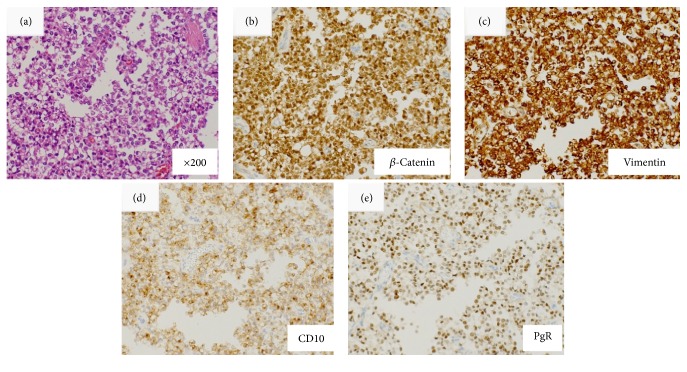
Histopathological results of biopsy samples obtained by endoscopic ultrasound-guided fine needle aspiration in Case  1. Histopathological analyses of the biopsy samples obtained by endoscopic ultrasound-guided fine needle aspiration in Case  1 are shown. (a) Hematoxylin-eosin staining revealed that the tumor cells exhibited acidophilic cytoplasm with small, round nuclei and formed pseudopapillary structures. Immunohistochemical analysis demonstrated that the tumor cells were positive for (b) *β*-catenin, (c) vimentin, (d) CD10, and (e) progesterone receptor.

Case  1 involved a 34-year-old male patient who was referred to our hospital for evaluation of epigastric pain. EUS revealed a well-encapsulated, smooth-surfaced, heterogeneous solid lesion at the body of the pancreas. The lesion measured 24 mm in diameter and contained hypoechoic/anechoic areas (Figure 1(A)). Hematoxylin-eosin staining of the biopsy specimens revealed that the tumor cells exhibited acidophilic cytoplasm with small, round nuclei and formed pseudopapillary structures. Immunohistochemical analysis revealed that the tumor cells were positive for *β*-catenin, vimentin, CD10, and progesterone receptor (Figure 2).

### 3.2. Case  2

Case  2 involved a 30-year-old male patient with acute abdominal pain. EUS showed a solid hypoechoic lesion in the tail of the pancreas. The lesion measured 20 mm in diameter and had a clear margin, regular borderline, and internal calcifications (Figure 1(B)). The pathological results were similar to those obtained by examination of the EUS-FNA samples in Case  1.

### 3.3. Case  3

Case  3 involved an 18-year-old male patient who was referred to our hospital for further evaluation of an abdominal mass incidentally detected by abdominal ultrasound. EUS showed a solid hypoechoic lesion at the body of the pancreas. The lesion measured 27 mm in diameter and contained small anechoic areas, a clear margin, and regular borderline (Figure 1(C)). The pathological results were similar to those obtained by examination of the EUS-FNA samples in Case  1.

The patients' clinical characteristics and EUS findings are shown in Table 1. The EUS findings were atypical but prompted us to consider the possibility of SPNs. However, other pancreatic tumors, especially neuroendocrine tumors (NETs), needed to be ruled out. Computed tomography (CT) in each case showed a solid tumor with slight enhancement at the margin, which is a not typical feature of SPNs (Figures 1(D)–1(F)). Magnetic resonance imaging (MRI) also did not provide additional useful information for achieving a definitive diagnosis. Consequently, we made a preoperative pathological diagnosis of SPN by EUS-FNA, and surgery was performed in all three patients. The final pathological diagnosis after surgery was an SPN with a Ki-67 labeling index of <2% in all three patients. All three patients remained free of disease during the follow-up period (Case  1, 26 months; Case  2, 12 months; Case  3, 18 months).

## 4. Discussion

An SPN is a very rare pancreatic tumor and is often recognized as a solid pancreatic mass. SPNs reportedly account for 1% to 2% of all pancreatic tumors [1]. They mostly affect young female patients; only 12.2% of patients with SPNs are male [2], most of whom are middle-aged. Therefore, SPNs in young male patients are extremely rare, and achieving a preoperative diagnosis of SPNs in our young male patients was difficult because previous data regarding typical imaging findings of SPNs are limited. We focused on the EUS findings of our three young male patients with relatively small SPNs and found that all SPNs were heterogeneous, hypoechoic, hypovascular, solid lesions.

Although most patients with SPNs have a good prognosis and favorable outcome with 5-year survival rates of almost 90% [1, 5], approximately 15% of SPNs have the potential for malignant transformation [5]. Specifically, one study showed that distant metastases occurred in 10% to 15% of patients with SPNs with lymph node metastasis in 2% [1]. The natural history of SPNs in male patients remains unclear, but it has been suggested that the biological malignant potential of SPNs is higher in males than in females [3, 4]. Therefore, surgical treatment should be considered when an SPN is diagnosed, especially in male patients. The number of patients diagnosed with SPNs has recently increased with the more widespread use of imaging modalities including ultrasound, CT, and MRI. These techniques have provided greater opportunities to incidentally detect relatively small lesions suspected to be SPNs.

Despite recent innovations in imaging modalities such as contrast-enhanced CT and MRI, differential diagnoses of pancreatic tumors are difficult to achieve, especially for relatively small lesions. Among several imaging modalities, EUS is generally accepted to be one of the best methods with which to detect and evaluate solid pancreatic lesions because of its high resolution [6]. Even by EUS, however, a definitive diagnosis of SPNs remains difficult to achieve for the following reasons. First, the EUS findings of SPNs are complex and often variable depending on the extent of hemorrhagic degeneration [7, 8]. Second, the EUS appearance of SPNs in male patients tends to be a small, solid lesion without a cystic component, and small SPNs frequently tend to present as pure solid masses with a sharp margin. Third, the EUS findings of SPNs of <3 cm seem to differ from those of SPNs of >3 cm [9]. All SPNs in the three young male patients in the present report were solitary, heterogeneous, hypoechoic, hypovascular, and solid on EUS. Besides these common features, small cystic changes were observed in Cases  1 and 3, while internal calcification was identified in Case  2. Differentiation of SPNs from NETs by radiological findings is generally difficult because both are sometimes accompanied by cystic degeneration and calcification, and even the pathological appearances may be similar between SPNs and NETs. We propose that the internal echo pattern could be the key EUS finding that is most useful for differentiating SPNs from NETs. It seems that a heterogeneous pattern is more prominent in SPNs than in NETs (Table 1), although further studies are needed to clarify this point.

After a lesion suspected to be an SPN has been identified by EUS, a definitive diagnosis of SPN must be made histologically. EUS-FNA is a diagnostic method that should be considered for this purpose because it is a well-established modality in the diagnosis of solid pancreatic tumors. The addition of EUS-FNA to currently used diagnostic modalities including CT or CT/EUS has been reported to significantly increase the diagnostic yield [10]. Consistent with this, a preoperative histological diagnosis of SPN could be made by EUS-FNA in all three cases in the present report.

In conclusion, we have herein presented three cases of relatively small SPNs in young male patients, mainly focusing on the EUS findings of these tumors. SPNs should be considered even in young male patients. EUS with EUS-FNA could be a useful diagnostic modality for SPNs, even in young males.

## Figures and Tables

**Table 1:** 

	Case 1	Case 2	Case 3
Age	34	30	18
Tumor size (mm)	24	20	27
Location	Body	Tail	Body
Echo level	Hypoechoic	Hypoechoic	Hypoechoic
Demarcation of tumor edge	Well-demarcated	Well-demarcated	Poorly demarcated
Internal echo pattern	Heterogenous	Heterogenous	Heterogenous
Cystic component	Presence	Absence	Presence
Calcification	Absence	Presence	Absence
Vascularity	Hypovascular	Hypovascular	Hypovascular
Dilation of MPD	Absence	Absence	Absence
Stenosis of MPD	Absence	Absence	Absence
Vessel invasion	Absence	Absence	Absence

MPD, main pancreatic duct.
